# Data for homogeneous thermofluorimetric assays for ethanolamine using aptamers and a PCR instrument

**DOI:** 10.1016/j.dib.2019.103946

**Published:** 2019-04-23

**Authors:** Mostafa Mahmoud, Stefan Laufer, Hans-Peter Deigner

**Affiliations:** a Furtwangen University, Institute of Precision Medicine, Jakob-Kienzle-Straße 17, 78054, Villingen-Schwenningen, Germany; b Department of Pharmaceutical and Medicinal Chemistry, Institute of Pharmaceutical Sciences, Eberhard Karls Universität Tübingen, Auf der Morgenstelle 8, 72076 Tübingen, Germany; c Fraunhofer Institute IZI, Leipzig, EXIM Department, Schillingallee 68, D-18057, Rostock, Germany

## Abstract

The data presented in this article describe the quantitative detection of small molecules e.g. ethanolamine through the shifts in the melting temperatures of aptamer beacons presented in the research article entitled “An aptamer based thermofluorimetric assay for ethanolamine” [1]. The data include prediction and optimization of the folding structure of the aptamers. Moreover, the data from using intercalating dyes such as SYBR green is included for comparison. The presented data could be used for the design of other small molecules sensing platforms using aptamers.

**Table:** Specifications table

Subject area	*Chemistry*
More specific subject area	*Analytical biochemistry*
Type of data	*Figures and graphs*
How data was acquired	*PCR LightCycler*^*®*^ *480, Roche*
Data format	*Analyzed*
Experimental factors	*The concentration of the aptamer beacon was fixed at 0.5 μM in all experiments except for the experiments with SYBR green (10x) and the corresponding melting analysis using aptamer beacons at the same concentrations (0.5, 1, 2 and 4 μM). The beacons were pipetted in a 96 wells PCR plate at a volume of 20 μL in ethanolamine binding buffer consisting of (20 mM TRIS, 100 mM NaCl, 0.02% Tween® 20 at pH 7.6).*
Experimental features	*The PCR machine (LightCycler*^*®*^ *480, Roche) was set to heat first till 99 °C (rate 4.4 °C/Sec) followed by holding for 5 minutes to ensure the complete denaturing of any DNA base pairings. Then, the fluorescence was measured from 95 °C to 20 °C with the SYBR Green filter (465 nm excitation and 510 nm emission) and a rate of 0.11 °C/Sec.*
Data source location	*Villingen-Schwenningen, Germany*
Data accessibility	*All data used and generated is included in this article and in its Supplementary Material*
Related research article	*M. Mahmoud, S. Laufer, H.P. Deigner, An aptamer based thermofluorimetric assay for ethanolamine, Biochimie, 158 (2019) 233–237.*

**Table:** 

**Value of the data** • The data could be used for optimizing homogeneous aptamer assays for small molecules • The data could give insights on aptamers thermodynamic and kinetic properties • The described methods are essential for simple and rapid detection of small molecules

## 1 Data

The obtained data show the optimization and validation of a thermofluorimetric assay for ethanolamine using ethanolamine binding aptamers and a PCR machine [1]. The assay was performed in a homogeneous format with no pre-activation of the aptamers and low volume of target (2 μL). The concentration of the aptamer beacon was fixed at 0.5 μM in all experiments except for the experiments with SYBR green (10×) and the corresponding melting analysis using aptamer beacons at the same concentrations (0.5, 1, 2 and 4 μM). The beacons were pipetted in a 96 wells PCR plate at a volume of 20 μL in ethanolamine binding buffer [4] consisting of (20 mM TRIS, 100 mM NaCl, 0.02% Tween^®^ 20 at pH 7.6). The analyte concentration of ethanolamine, phenylethylamine, ethanol and propylamine was varied from (5–100 nM) and were added in a volume of 2 μL per well. Additionally, 1 μM concentration of phenylethylamine, ethanol and propylamine was tested.

### 1.1 Structure prediction and optimization of the aptamers folding

**Fig. 1:**
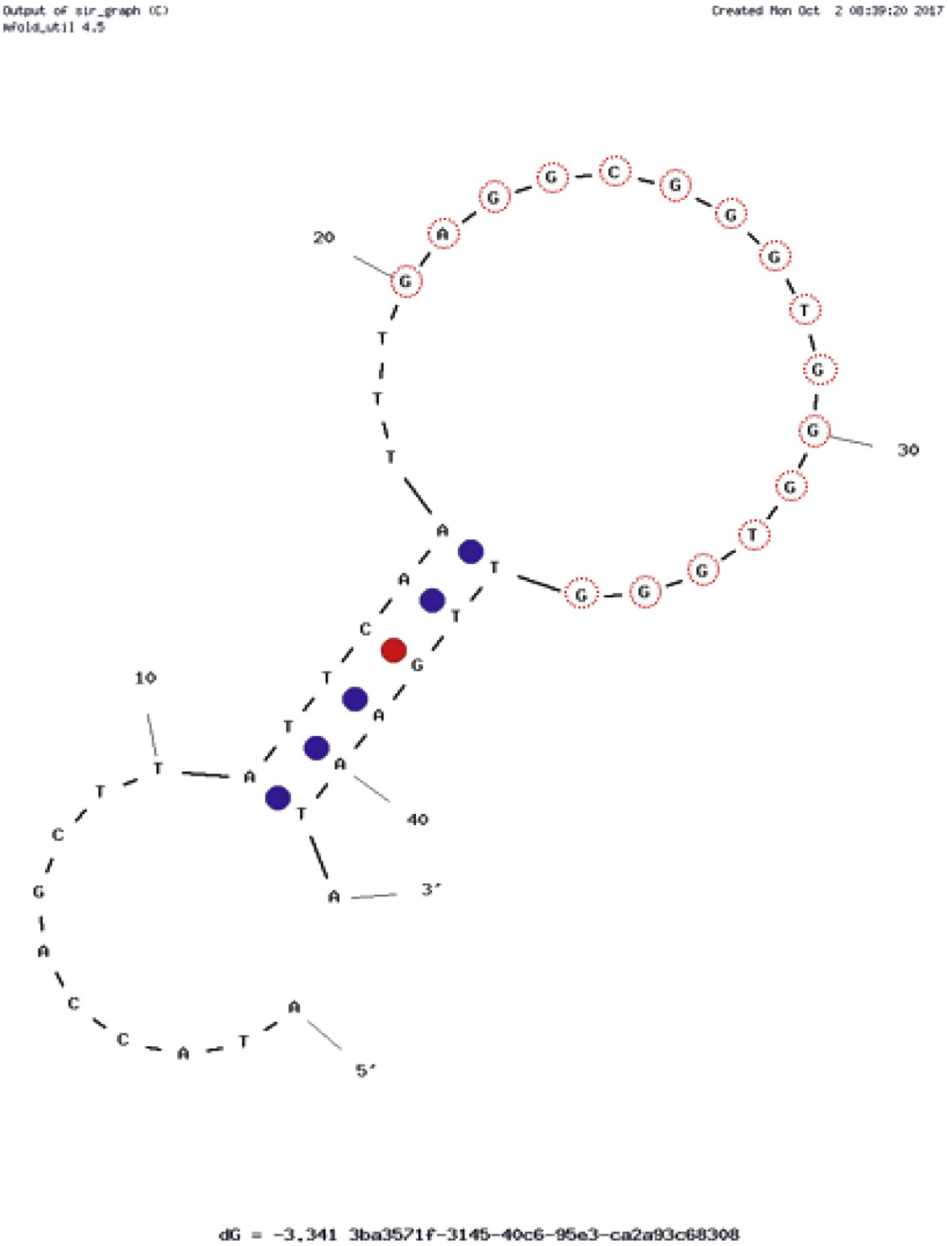
The predicted secondary structure of the ethanolamine aptamer 42 nucleotides (EA#14.3K42) [4] a truncated version of the original 96nt (EA#14.3) [5] aptmaer. A fluorophore (Fluorescein) was added to the 5′ end and a quencher (DABCYL) to the 3′ end to produce a beacon. Folding simulated using mfold [2]. The G-rich consensus sequence is marked by red circles.

**Fig. 2:**
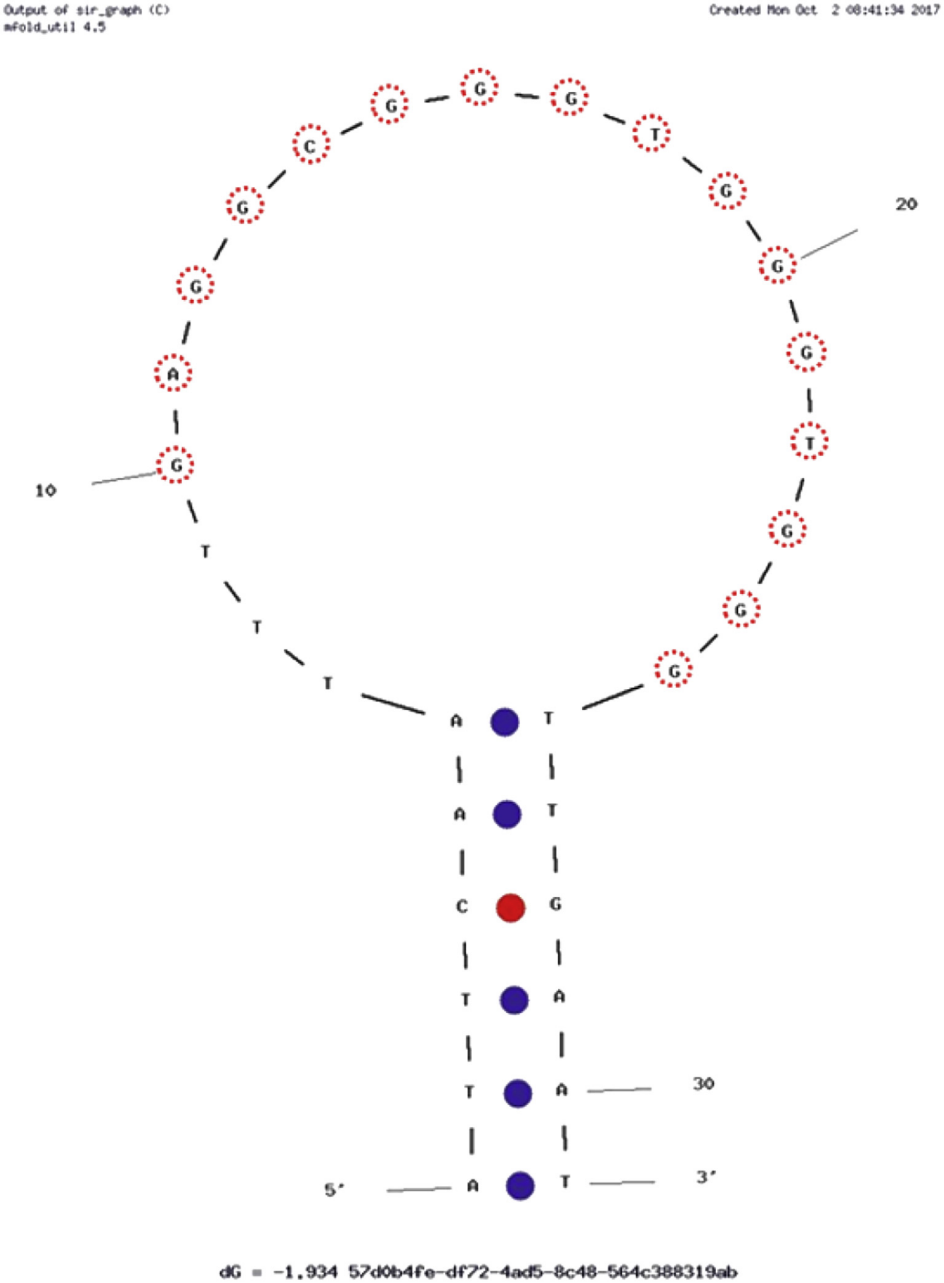
The predicted secondary structure of the modified ethanolamine aptamer EA#14.3K31 a truncated version of the EA14.3K42 aptamer. A fluorophore (Fluorescein) was added to the 5′ end and a quencher (DABCYL) to the 3′ end to produce a beacon. Folding generated using mfold [2]. The G-rich consensus sequence is marked by red circles.

**Fig. 3:**
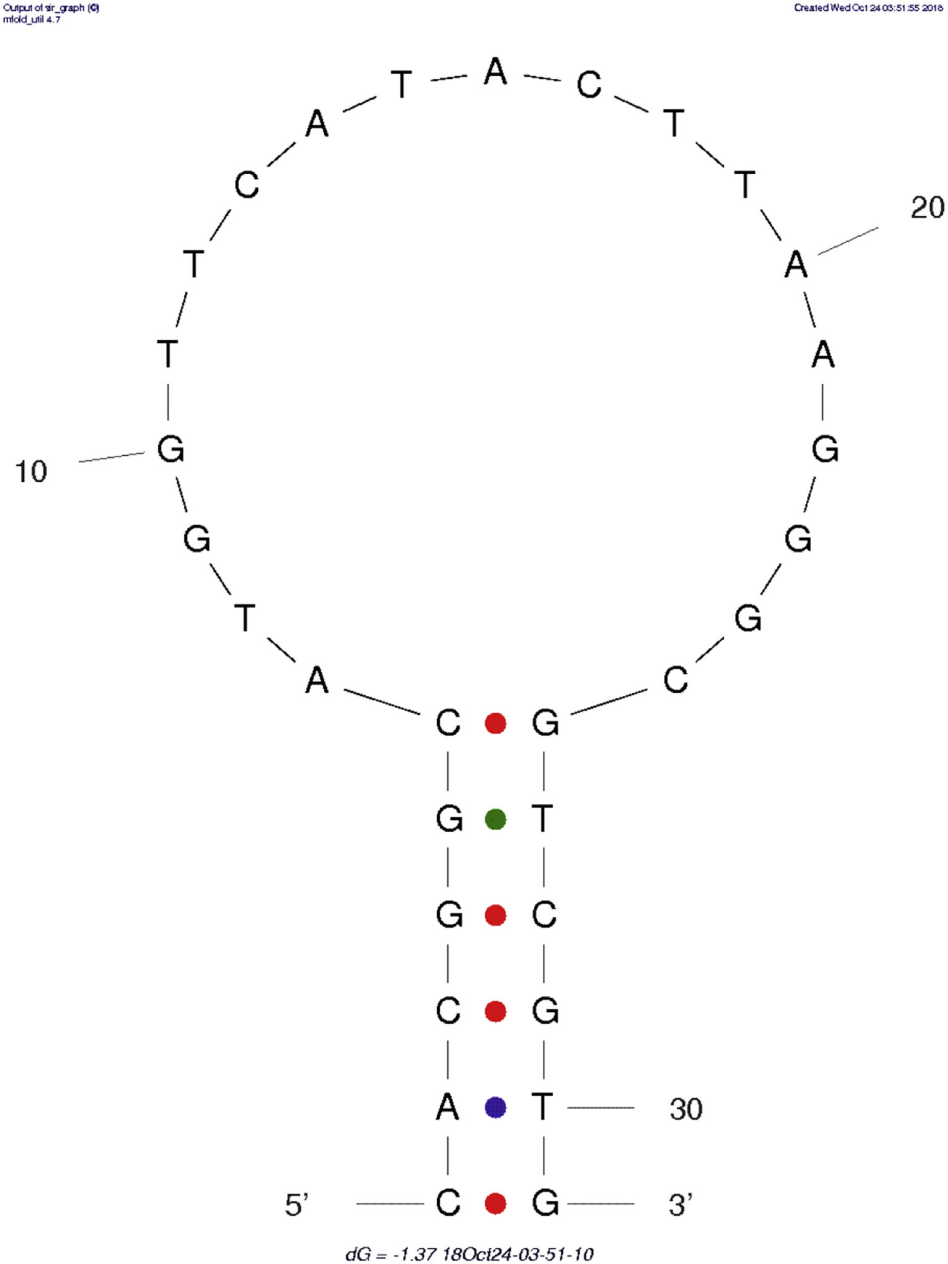
The predicted secondary structure of the scrambled aptamer used as negative control for the ethanolamine 31 nucleotides modified aptamer. A fluorophore (Fluorescein) was added to the 5′ end and a quencher (DABCYL) to the 3′ end to produce a beacon. Folding generated using mfold [2].

The aptamer folding structure in the given buffer conditions was predicted using mfold [2]. The aptamer 42 nucleotides EA#14.3K42 [3] (Fig. 1) did not show optimal stem loop folding. Therefore, the sequence was modified by truncation to produce a 31-nucleotide aptamer EA#14.3K31 (Fig. 2). The G-rich binding sequence [4] was conserved during this truncation. A scrambled sequence consisting of 31 nucleotides showing a secondary structure similar to the ethanolamine aptamer was added as a non-binding aptamer (Fig. 3). The aptamers were then modified with A fluorophore (Fluorescein) on the 5′ end and a quencher (DABCYL) on the 3′ end to produce a beacon [3].

### 1.2 Melting profiles and thermofluorimetric analysis

**Fig. 4:**
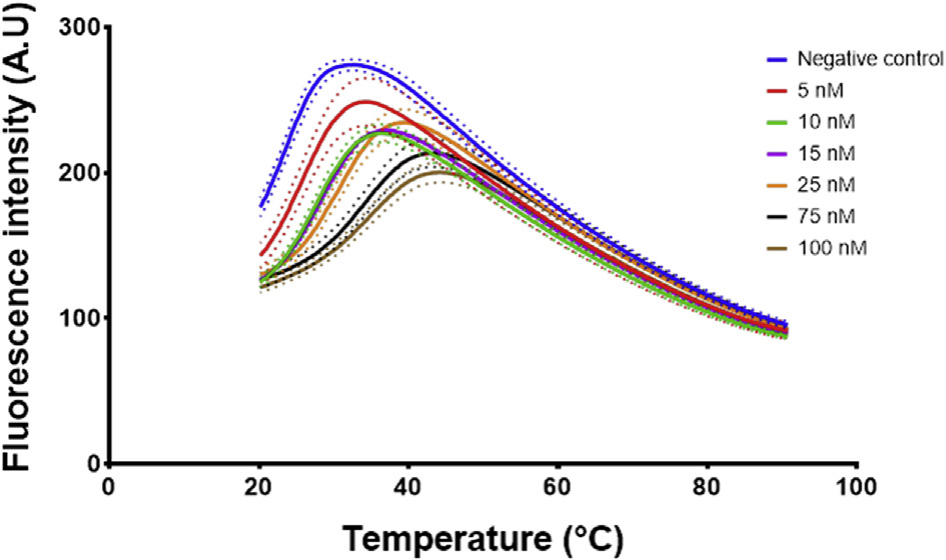
Signal data from the qPCR machine showing fluorescence intensity against temperature °C. The lines represent the average of at least 4 wells, the dots represent the SD. Each well had 20 μL of ethanolamine aptamer EA14.3K42 at 0.5 μM concentration. 2 μL of ethanolamine (0–100 nM) were added to each well before measuring in the PCR.

**Fig. 5:**
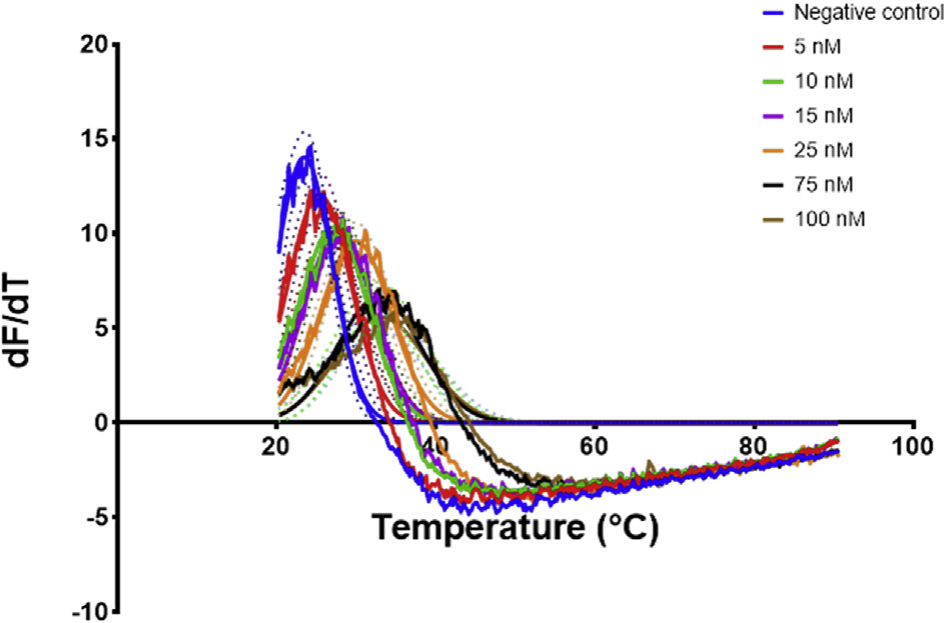
The first derivative of the signal measured over temperature obtained from Fig. 4. The curve was generated using graphpad prism First derivative with 2nd order smoothing (4 neighbours) then Model (Gaussian distribution) Y=Amplitude*exp(-0.5*((X-Mean)/SD)ˆ2) was fitted to the data points and the mean defined the Tm, the Tm was then plotted against the concentration to produce the calibration curve. The lines represent the fitting and the dots represent the 95% confidence bands of the fit.

**Fig. 6:**
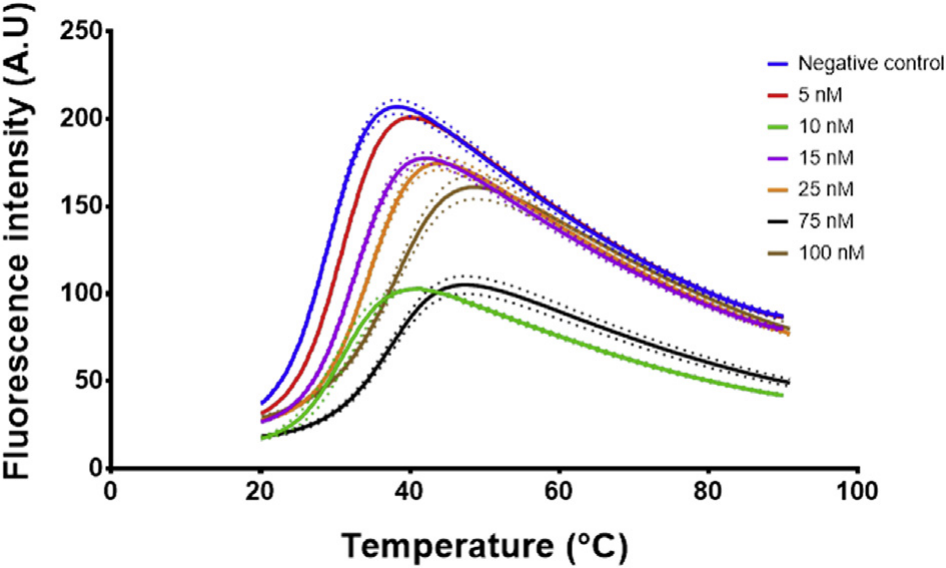
Signal data from the qPCR machine showing fluorescence intensity against temperature °C. The lines represent the average of at least 4 wells, the dots represent the SD. Each well had 20 μL of ethanolamine aptamer EA14.3K31 at 0.5 μM concentration. 2 μL of ethanolamine (0–100 nM) were added to each well before measuring in the PCR.

**Fig. 7:**
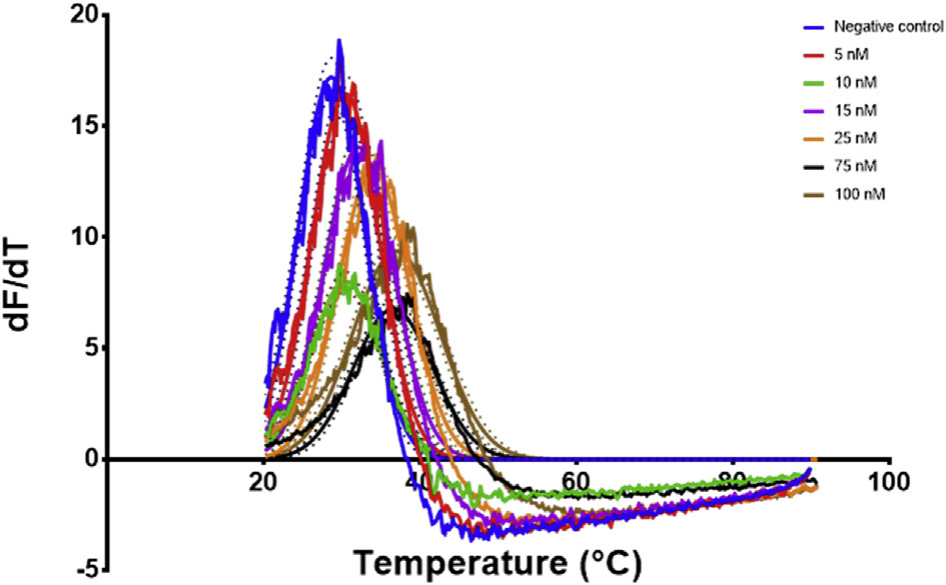
The first derivative of the signal measured over temperature obtained from Fig. 6. The curve was generated using graphpad prism First derivative with 2nd order smoothing (4 neighbours) then Model (Gaussian distribution) Y=Amplitude*exp(-0.5*((X-Mean)/SD)ˆ2) was fitted to the data points and the mean defined the Tm, the Tm was then plotted against the concentration to produce the calibration curve. The lines represent the fitting and the dots represent the 95% confidence bands of the fit.

**Fig. 8:**
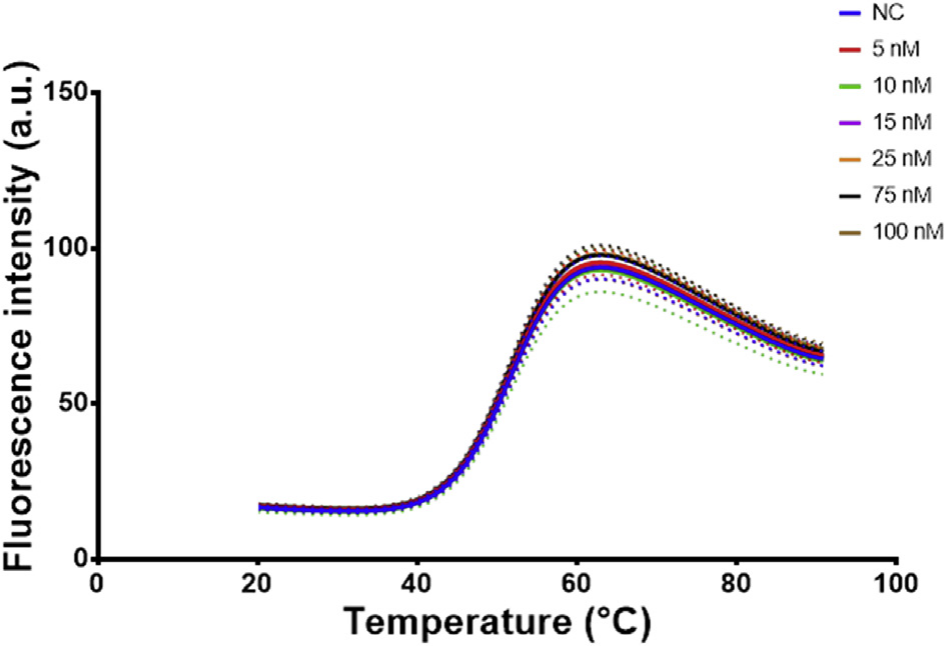
Signal data from the qPCR machine showing fluorescence intensity against temperature °C. The lines represent the average of at least 4 wells, the dots represent the SD. Each well had 20 μL of scrambled ethanolamine aptamer sequence at 0.5 μM concentration. 2 μL of ethanolamine (0–100 nM) were added to each well before measuring in the PCR.

**Fig. 9:**
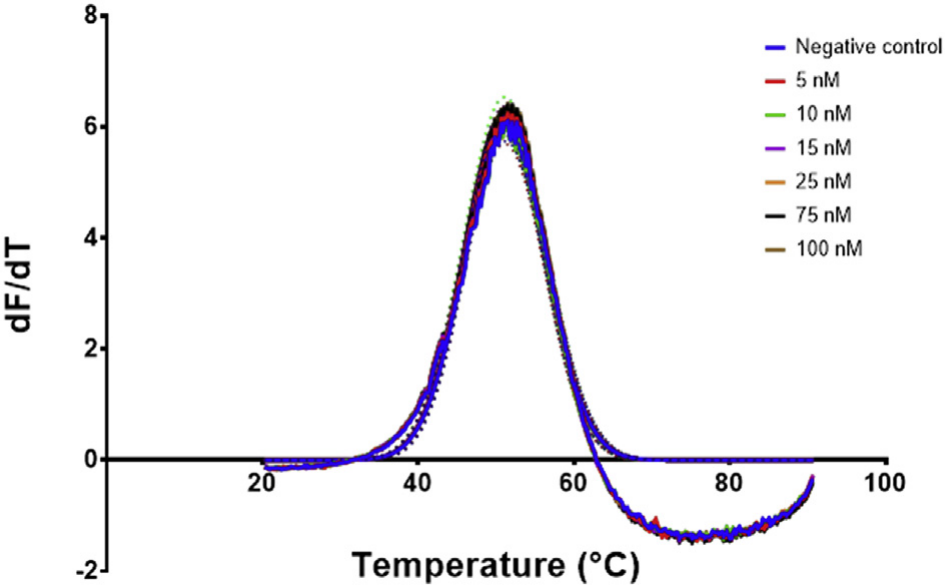
The first derivative of the signal measured over temperature obtained from Fig. 8. The curve was generated using graphpad prism First derivative with 2nd order smoothing (4 neighbours) then Model (Gaussian distribution) Y=Amplitude*exp(-0.5*((X-Mean)/SD)ˆ2) was fitted to the data points and the mean defined the Tm, the Tm was then plotted against the concentration to produce a calibration curve. The lines represent the fitting and the dots represent the 95% confidence bands of the fit.

The three aptamers EA#14.3K42, EA#14.3K31 and the scrambled sequence were prepared in the ethanolamine binding buffer [4] in a 96 well PCR plates at a volume of 20 μL per well. Afterwards, the sample was added to the wells at a volume of 2 μL per well and directly measured in the PCR machine. The obtained data from the PCR machine for each plate was averaged (Fig. 4, Fig. 6, Fig. 8) and then the first derivative with second order smoothing (4 neighbours) was produced (Fig. 5, Fig. 7, Fig. 9). Then the Model (Gaussian distribution) Y=Amplitude*exp(-0.5*((X-Mean)/SD)ˆ2) was fitted to the data points and the mean defined the Tm.

**Fig. 10:**
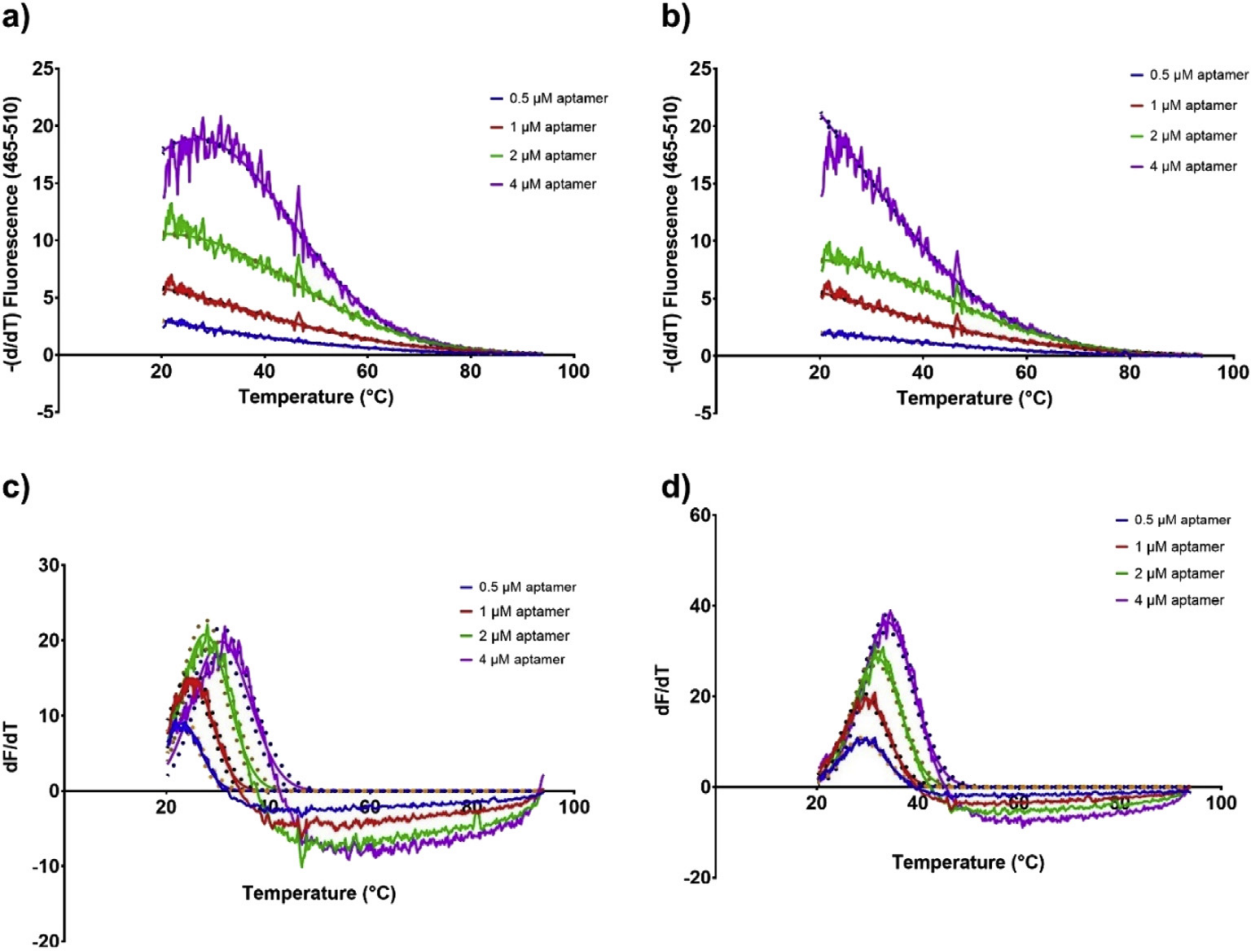
a) melting profile of different concentrations of EA#14.3K42 aptamer using 10× SYBR green b) melting profile of different concentration of the EA#14.3K31 aptamer using 10× SYBR green c) melting profile of different concentrations of EA#14.3K42 aptamer beacon d) melting profile of different concentrations of the EA#14.3K31 aptamer beacon. The lines are the fit using the Model (Gaussian distribution) Y=Amplitude*exp(-0.5*((X-Mean)/SD)ˆ2) and the dots represent the 95% CI of the fit. The aptamer beacons show sharper well-defined peaks in comparison to the use of the SYBR green.

Similarly, SYBR green a DNA intercalating dye [6] was used at 10× concentration and a varying amount of the non-labelled aptamers (0.5, 1, 2 and 4 μM) was used to capture the melting profile (Fig. 10a and b). The data obtained was handled slightly different and the negative first derivative was produced for the SYBR green experiments. These results were compared to the melting profiles of aptamer beacons at the same concentrations (0.5, 1, 2 and 4 μM) (Fig. 10c and d).

### 1.3 Specificity of the assay and ethanolamine binding response

**Fig. 11:**
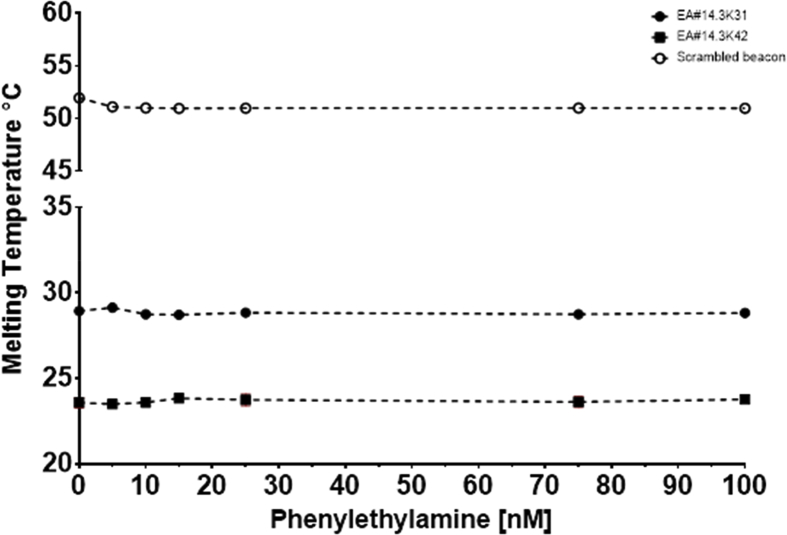
Melting temperature °C plotted against the concentration of phenylethylamine. The points represent the average of at least 3 experiments with on plate redundancy of 3 wells. The error bars represent the standard deviation, some error bars are smaller than the symbol size. Two aptamer beacons were used the EA#14.3K42 is 42nt whereas the EA#14.3K31 is 31nt and a scrambled sequence as a no binding control.

**Fig. 12:**
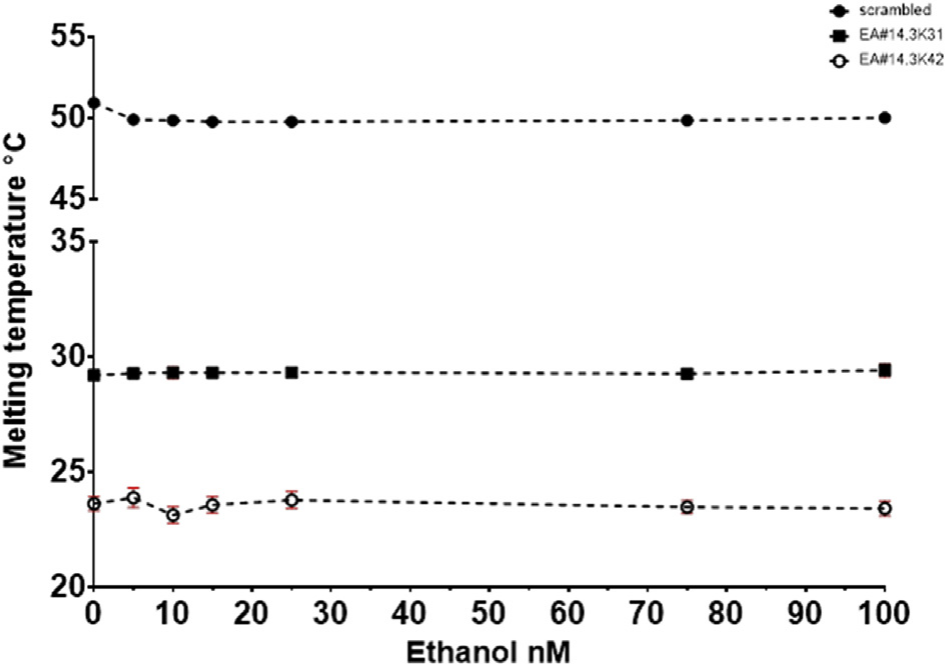
Melting temperature °C plotted against the concentration of Ethanol. The points represent the average of at least 2 experiments with on plate redundancy of 3 wells. The error bars represent the standard deviation, some error bars are smaller than the symbol size. Two aptamer beacons were used the EA#14.3K42 is 42nt whereas the EA#14.3K31 is 31nt and a scrambled sequence as a no binding control.

**Fig. 13:**
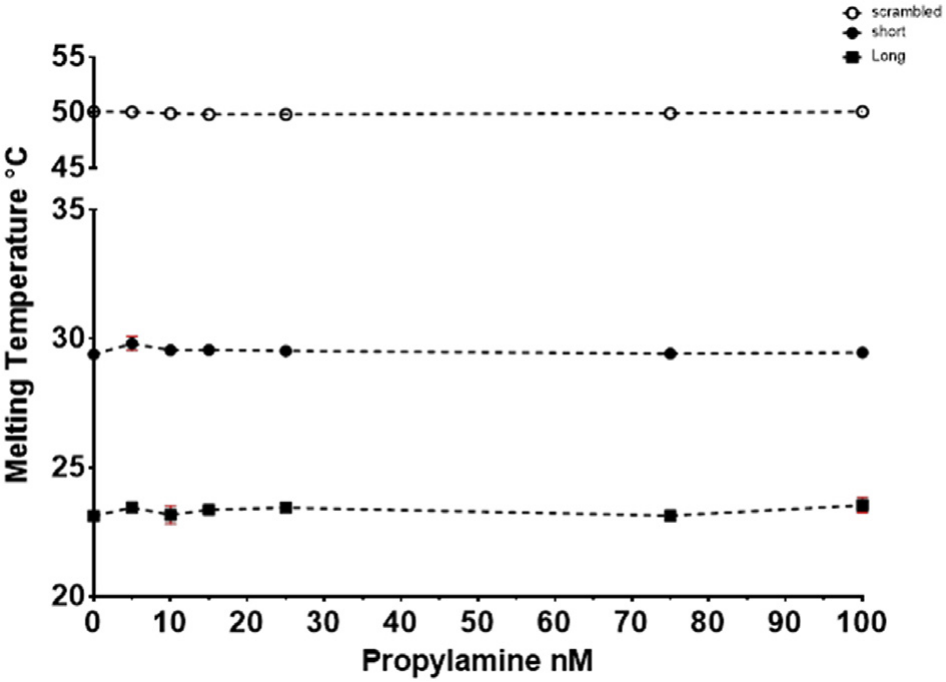
Melting temperature °C plotted against the concentration of Propylamine. The points represent the average of at least 2 experiments with on plate redundancy of 3 wells. The error bars represent the standard deviation, some error bars are smaller than the symbol size. Two aptamer beacons were used the EA#14.3K42 is 42nt whereas the EA#14.3K31 is 31nt and a scrambled sequence as a no binding control.

To further verify the specificity of the response of the ethanolamine aptamers, the response to various structurally similar compounds e.g. Phenyethylamine, ethanol and propylamine was tested. The obtained melting temperatures from the first derivative fit was plotted against the corresponding concentrations of the analyte to produce the calibration curves (Fig. 11, Fig. 12, Fig. 13).

**Fig. 14:**
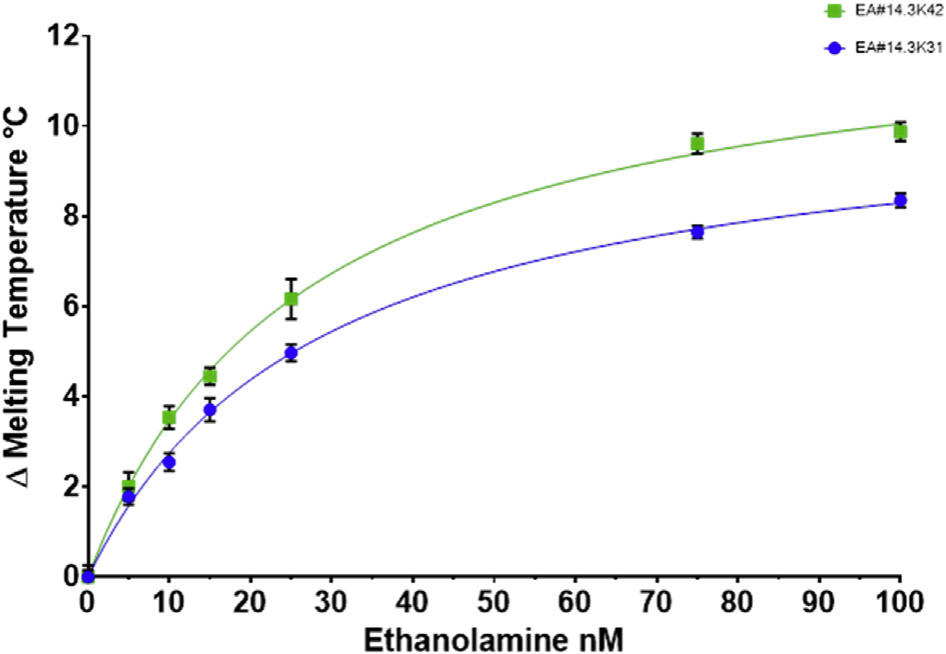
ΔTm °C plotted against the concentration of ethanolamine. The points represent the average of at least 4 experiments with on plate redundancy of 4 wells. The error bars represent the standard error of the mean, some error bars are smaller than the symbol size. Two aptamer beacons were used the EA#14.3K42 is 42nt whereas the EA#14.3K31 is 31nt. The lines represent the best fit using one site binding equation Y=Bmax*X/(Kd + X).

The kinetic parameters were determined using the one site binding equation Y=Bmax*X/(Kd + X). The melting temperatures were background corrected and plotted against the concentration of the ethanolamine to produce the curve (Fig. 14).

## 2 Experimental design, materials and methods

### 2.1 Materials

Aptamer beacons (with 5′ Fluorescein and 3′ DABCYL) were synthesized and purified by Integrated DNA Technologies (Coralville, IA). Ethanolamine, phenylethylamine, oxytetracycline and SYBR^®^ Green I (10,000× in DMSO) were purchased from Sigma-Aldrich (Darmstadt, Germany).

The following are the sequences of the used aptamers all of which had 5′ Fluorescein and 3′ DABCYL and with no labels for use with SYBR green.

Ethanolamine binding aptamer EA#14.3K42: ATACCAGCTTATTCAATTTGAGGCGGGTGGGTGGGTTGAATA.

Ethanolamine binding aptamer EA#14.3K31: ATTCAATTTGAGGCGGGTGGGTGGGTTGAAT.

Scrambled sequence used as ethanolamine negative: CACGGCATGGTTCATACTTAAGGGCGTCGTG.

### 2.2 Methods

#### 2.2.1 Melting temperature profiles

To account for signal variation and scattering of the data, the on-plate redundancy was at least 4 identical wells per parameter. Additionally, each experiment was repeated at least 3 times to assess both reproducibility and inter-assay variation.

The concentration of the aptamer beacon was fixed at 0.5 μM in all experiments except for the experiments with SYBR green (10×) and the corresponding melting analysis using aptamer beacons at the same concentrations (0.5, 1, 2 and 4 μM). The beacons were pipetted in a 96 wells PCR plate at a volume of 20 μL in ethanolamine binding buffer [25] consisting of (20 mM TRIS, 100 mM NaCl, 0.02% Tween^®^ 20 at pH 7.6).

The analyte concentration of ethanolamine, phenylethylamine, ethanol and propylamine was varied from (5–100 nM) and were added in a volume of 2 μL per well. Additionally, 1 μM concentration of phenylethylamine, ethanol and propylamine was tested.

#### 2.2.2 PCR protocol

The PCR machine (LightCycler^®^ 480, Roche) was set to heat first till 99 °C (rate 4.4 °C/Sec) followed by holding for 5 minutes to ensure the complete denaturing of any DNA base pairings. Then, the fluorescence was measured from 95 °C to 20 °C with the SYBR Green filter (465 nm excitation and 510 nm emission) and a rate of 0.11 °C/Sec.

#### 2.2.3 Data analysis

Data analysis (plotting and fitting using one site binding equation) was performed using GraphPad Prism version 7.00 for Windows, GraphPad Software, La Jolla California USA, www.graphpad.com and JMP^®^, Version *<13.1>*. SAS Institute Inc., Cary, NC, 1989–2007. The raw data obtained from the PCR (Fig. 4, Fig. 6, Fig. 8) was used to generate first derivative graphs for each individual experiment (Fig. 5, Fig. 7, Fig. 9). These first derivatives were fitted using a Gaussian distribution with the following equation Y=Amplitude*exp(-0.5*((X-Mean)/SD)ˆ2.
