# Supplementary material for: Data for homogeneous thermofluorimetric assays for ethanolamine using aptamers and a PCR instrument
Source: Data Brief. 2019 Apr 23;24:103946. doi: 10.1016/j.dib.2019.103946 (PMC6506705; doi:10.1016/j.dib.2019.103946)
Supplement: Multimedia component 1 [file mmc1.docx]

Hochschule Furtwangen (HFU)

Prof. Dr. Hans-Peter Deigner

Jakob-Kienzle Str. 17

DE-78054 Villingen Schwenningen

**Author agreement**

**Manuscript submission to Data in Brief: Data for homogeneous thermofluorimetric assays for ethanolamine using aptamers and a PCR instrument**

Dear Editor,

All authors contributed significantly and agree to submission and publication.

We have no conflict of interest to disclose.

We have adjusted the manuscript according to the referees’ comments

Thank you for consideration.

Best regards,

Prof. Dr. Hans-Peter Deigner	20/03/2019 Villingen-Schwenningen
